# 

**Published:** 1916-01-08

**Authors:** 


### CONTENTS.

| | PAGE |
|---|---|
| **The Treatment of Wounds Controversy** ... | 311 |
| **The Tubercular Patients Problem** ... ... | 312 |
| **Hospital and Institutional News** ... ... ... | 313 |
| The New Year's Honours—Navy and Army Honours—Norwich Secretaryship Again Vacant—The Defender of Chitral—Mr. Carnt's Departure from Manchester—The Attendance at Annual Meetings—Red Cross Hospitals and Sunday Cinematograph Shows—On Cutting Down the Annual Report—Cerebro-Spinal Meningitis—An Encouraging Example—"The Boys in Blue"—How Bath is Tackling the Measles Problem—The Trying Time of the Surgical Aid Society—The Northern Miners' Ambulances—The Order of St. John—Wastage Among Austro-German Doctors — Dundee's New Hospital for Infants—Extra Work and Its Reward in Hospital Offices—A British Ambulance in Italy—British Hospitals for French Wounded—The *Gaulois'* List—The Perth Public Hospital, Western Australia—Anthrax and the Shaving-Brush—Non-Panel Doctors and Insured Patients—Anti-Typhoid Inoculation—Sisters as Anæsthetists—This Week's Drug Market. | |
| **The Treatment of Abdominal Wounds** ... | 319 |
| The Contrasts of Two Campaigns. | |
| **A Revival in Antiseptics** ... ... ... ... | 320 |
| Two Interesting Papers. | |
| **The Vanquishing of Ophthalmia** ... ... ... | 321 |
| A Crusade against One of the Plagues of Egypt. | |
| **The Finance of a Year's Red Cross Work** ... | 323 |
| The Administration of Two Millions of Money. | |
| **National Insurance Act Developments** ... ... | 325 |
| A General Survey of Developments during the Year 1915. | |
| **Textile Supplies for Hospitals** ... ... ... | 327 |
| II.—The Basis of Values. | |
| **Hospitals and the Poets** ... ... ... ... | 329 |
| The Poet Laureate, Henley, and Two Famous Hospitals. | |
| **The Department of Modern Nursing** ... ... | 330 |
| St. Mary's Hospital for Women and Children, Plaistow. | |
| **The Institutional Library** ... ... ... ... | 331 |
| Diseases of Nutrition and Infant Feeding—Diseases of the Nervous System—In a French Military Hospital—With the Turkish Army in the Crimea and Asia Minor—How to Become a Nurse—Women in the Public Health Service—The Secret of Human Power. | |
| **Parliament and the Profession** ... ... ... | 333 |
| **Passing Events and Topics** ... ... ... ... | 333 |
| **Round the World** ... ... ... ... ... | 334 |
| **The Institutional Worker** ... ... (Suppl.) | 1 |
| A Charity Appeal in War Time: Answers to December Question. | |

